# Evaluation of new approaches to the access of official monitoring results for live bivalves molluscs

**DOI:** 10.1093/eurpub/ckz018

**Published:** 2019-02-27

**Authors:** Manuel Beja da Costa, Magdalena Jurczuk, Bernardo Marques, João Nuno Silva

**Affiliations:** 1 INESC-ID Lisboa, Instituto Superior Técnico, Universidade de Lisboa, Lisboa, Portugal; 2 Tulane University School of Public Health and Tropical Medicine, New Orleans, Louisiana, USA; 3 Instituto Superior Técnico, Universidade de Lisboa, Lisboa, Portugal

## Abstract

**Background:**

Live bivalve molluscs, echinoderms, tunicates and marine gastropod are referred in EU food laws, and require member states to implement official controls in classified production areas, with the monitoring and classification of those areas. If, due to contaminant tests results, a production area is closed, any product from there is prohibited to be commercialized. Mobile applications optical character recognition (OCR) functionalities could ease the access to contaminant levels and production area classifications. This study verifies what information is available in live bivalves’ labels, describes an OCR algorithm for those labels and evaluates it.

**Methods:**

86 labels were selected from four sale points in Lisbon, and photographed using smartphones. Each label was evaluated by a human to determine what data was available (either required or not). An OCR algorithm was developed and applied on the collected labels and validated against the data extracted by the human analysis.

**Results:**

The analysis shows that all the labels included the required information, and 63% of the labels included the identifier for the production zone. The label-reading algorithm performs with an accuracy of 79.85% for the individual values.

**Conclusion:**

High accuracy of the developed label-reading algorithm shows potential for providing instant automatic access to the date and production area, but is affected by the variability on the label structure. Although not required by food laws, the majority of the sampled labels included complementary information (classified production area) that will allow access to more precise information about the existing biotoxin tests and analysis results.

## Introduction

Live bivalve molluscs is a popular ingredient in gastronomical traditions throughout Europe and have an important impact on the European economy.^1^^,^^2^ These shellfish however, are vulnerable to exposure to diverse contaminants that can pose health risks^3^ to consumers. Due to the frequently microbiological and chemical hazards being reported, the European food laws define a series procedures and rules on the hygiene of foodstuffs.

For instance, with respect to live bivalves Regulations 853/2004^4^ and 854/2004^5^ demand each member state to: (i) define the geographical delimitations of the production areas, (ii) classify these areas based on contamination levels and associated risks, (iii) regulate water quality test procedures and (iv) regulate the monitoring of the process from production to consumption. The legal limits for the various live bivalves contaminants are also defined in European regulations for Marine Biotoxins,^4^^,^^6^ Metals,^7^ Polycyclic aromatic hydrocarbons^7^ and Microbiological.^5^^,^^8^^,^^9^ If the levels of a series of tests and analysis of some contaminants are above the permitted limits or suggest a risk to human health, the production area must be reclassified or shut down and food business operators must ensure that any products recently produced in that area are not traded, sold or consumed. At the moment dispatch centres and processing establishment have access to this information^5^ and can cross reference it with the production date and area from the registration documents.^4^ When live bivalves leave dispatch centres and enter the retail circuit the labels do not need to include the production areas.^10^

Although the classification of production areas should be immediately communicated to the relevant parties^5^ (such as producers and operators of purification or dispatch centres), access to this information by the public and other participants in the retail and commercialization can be facilitated and optimized by the use of a mobile application. Such mobile application, included in the *mHealth*^11^^,^^12^ class of applications, would improve access to contaminant tests results, production areas classifications and other food safety related information. For example, the Food Safety Mobile Application^13^ from the Centre for Food Safety of Hong Kong allows users to obtain and share general food safety information in a ubiquitous and straightforward way. Besides general information about food safety, this application also presents food related alerts, recalls and prohibitions.

A similar *mHealth* application can be developed to allow users to access bivalves’ toxicity results, commercialization authorizations and general information. This application would benefit private consumers, commercial entities and inspection authorities, providing accurate information in a convenient way.

The work here described is threefold, (i) evaluates if the labels include all the information necessary for the correct assessment of production/commercialization authorization, (ii) determines if an automatic label-reading algorithm can accurately process and decode the available information and (iii) discusses the current quality of labels and the applicability mobile application and automatic label reading mechanism to ease access to bivalves safety information.

## Methods

A set of 86 labels was collected in Lisbon, Portugal in in four fish markets and supermarkets between the months of July 2017 and January 2018. Each location was visited every 2 weeks, but due to the stock availability and authorizations, in some visits no samples were collected.

Two different smartphones, iPhone 6S and Samsung J7, were used to photograph the labels using the standard settings and without any special lighting or photo preparation. The use of different devices exposed the algorithm to different image resolutions to assure its robustness to the variability of devices expected in real use scenario.

After the collection of labels two different analyses were made, as described in the next sections.

### Label human-based analysis

All 86 labels were manually decoded and studied for completeness of information, and a table was created detailing the following variables for each label:

- species;
- date of packaging;
- classified production area;
- production method (aquaculture or wild harvest), and
- country of production
- purification information.

Some of this data is not required in the label, but can be used by the consumer to access complementary information about the product.

This table is also used to validate the results obtained from the algorithm’s readings and calculate its accuracy.

Each label was characterized as: ‘complete’^10^ if the required data is present in the label and ‘extra’ if non-required data that could be used in the mobile application was available. This extra data will allow the mobile application/user to access information about the contamination levels and classification for the specific production area.

### Label recognition algorithmic-based analysis

An automatic character recognition, data extraction and label recognition algorithms were developed to try to extract the relevant information from the bivalves labels.

**Figure 1:**
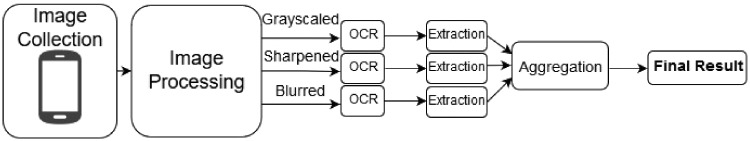
Architecture of the label recognition algorithm

After multiple experiments the best architecture for this software system is presented in figure 1.

After the collection of the labels (‘image collection’ step on figure 1), the first stage of the algorithm (‘image processing’) is started. To optimize next processing stages and reduce influences of camera capture quality and label faults, three intermediate images are produces, each resulting from the application of a different image processing techniques to the original photo: (i) conversion of original image to greyscale; (ii) application of a 3 × 3 kernel sharpening filter^14^ to the original image and (iii) application of a 3 × 3 kernel blurring filter.^14^ The sharpening filter improves the reading accuracy on poorly focussed images, while the blurring filter’s adaptive thresholding makes the algorithm robust to shadows.

This image processing step produced the outputs in the form of three distinct images that were independently processed in the second stage of the algorithm by the optical character recognition (OCR) module.

This ‘OCR’ module was developed using the Tesseract Python^15^ library that implements the Tesseract^16^ algorithm. This library source code and necessary training data is publicly available in the open source community. To further optimize the produced output, Tesseract was configured to recognize only the Portuguese alphabet and to allow sparse text. The adjustment for Tesseract to read sparse text was necessary to increases accuracy when reading text with a non-standardized structure, as is the case with the bivalve labels sampled in this study.

The third stage (‘extraction’ module) performs the data extraction from the text returned from the OCR, using pattern matching and statistical analysis. Since the labels included information that were not essential for the presented study, some of the text that was read by the OCR module was irrelevant and ignored. Furthermore, some of the text recognized in the OCR stage did not exactly match the expected information because when Tesseract is incapable of recognizing the full word, an approximated result was produced. These two factors required an additional processing on the ‘Aggregation’ module. This module estimates an approximation to the real data, by comparing the text returned by the ‘extraction’ module with the expected data formats (in the case of dates) and values (in the case of all other data). The algorithm was programmed to compare and match the OCR returned text with the following:

- list with all species, in Portuguese and in Latin;
- list with all classified production areas from Portugal and Galicia;
- list with all possible production methods;
- list with all possible countries of production;
- Purification information
- date format with the ‘/’, ‘-’ and ‘.’ separators;

By matching the returned text from each processed image with the expected text for each variable, and using proximity formulas, each processed image was assigned a fixed set of values for each previous variable.

The values of the variables (associated with an accuracy) of each intermediate image (greyscaled, sharpened and blurred) was merged to extract the best one: the value with the highest accuracy was chosen as the final value and assigned to the original photo ‘Final Results’.

Obtaining the final result for the date variable differed from the other variables because the date recognition algorithm did not returned an accuracy ratio, but instead a specific set of digits. A voting system was implemented on which, each variation of the original image voted on a day and a month. The ones with the most votes were chosen as the ‘Combined Result’.

Since the packaging date does not need to include the year, the algorithm was programmed to assume the current year as the year of packaging, with the exception of the case where the day/mount value corresponds to a date in the future (for instance a label with a December date being read in January).

After the automatic processing of all the labels and extraction of final results, this data was validated against the results of the human analysis to calculate the algorithms accuracy.

## Results

The dataset was composed by 86 labels. These labels referred the following species: *Pharus legumen, Ensis spp, Donax* spp*, Spisula solida, Ruditapes philippinarum, Ruditapes decussata, Cerastoderma edule, Crassostrea* spp and *Mytilus* spp. These products were originated from: Portugal (69.8%), Spain (25.6%) and the Netherlands (4.6%). More than 30% of the samples corresponded to bivalves produced outside of Portugal.

### Human analysis

Table 1 presents the results of human analysis of the labels. All labels were considered ‘complete’, since they included the required information.^10^ In total 63% of the labels also included the classified production area and 16% mentioned that the product received treatment in a purification centre.

**Table 1:** Evaluation of labels information

		With classified production area			Without classified production area				
		Purification information			Purification information			Extra Information	
	Total	yes	No	Total	Yes	No	Total	Yes	No
**Portugal**	60	2	40	42	5	13	18	47	13
**Spain**	22	1	10	12	4	6	10	16	6
**Netherlands**	4	–	–	–	–	4	4	–	4
**Total**	86	3	50	54	9	23	32	64	23

A total of 74% of the labels contained data that a consumer/mobile application user can use to retrieve information about the product: either the packaging gate and classified production area, or the mention to the product having received treatment in a purification centre.

Comparing the labels from different production countries, it is possible to observe that 70% of the samples produced in Portugal included the classified production area, while from Spain only 54% of the label included that information. None of products from Netherlands included the production area. All Spanish products that included the production area came from Galicia.

### Algorithm results

Table 2 shows the results obtained from each individual OCR algorithm (greyscale, sharpened and blurred) and from the final data extraction and aggregation, and presents for the available data the amount of correctly read values. Each filter overcame different problems and issues in the original image, and for every image condition each of the filters was better than the other two.

**Table 2:** Accuracy of the reading algorithm

	Greyscaled [%]	Sharpened [%]	Blurred [%]	Final/combined [%]
**Species**	80.24	74.41	84.88	88.37
**Date**				
** Components**	64.34	55.04	56.97	69.38
** Whole**	41.86	30.23	32.56	50.00
**Zone**	70.93	68.60	74.41	82.55
**Type of production**	52.32	60.46	54.65	73.00
**Country**	72.00	82.55	72.00	84.88
**Purification**	91.86	94.18	89.53	95.34
**Overall accuracy (using date components)**	68.99	68.79	68.81	79.85

This final result is more precise that the best partial results, because for every label this value was calculated using a proximity heuristic that correlates and aggregates the partial results into one final ‘combined’ result.

Table 2 shows that the ‘date’ field has the lowest accuracy. The ‘Date—Components’ row expresses the overall reading accuracy of the two individual components of a date (day and month), whereas the ‘Date—Whole’ row expresses the accuracy when reading of a complete date (the two components must be correct). The year was inferred from the current date and the month presented in the label.

Some labels included information stating that the product had been subject to a purification process. The OCR algorithm was programmed to match such statement returning whether the sample had been subject to purification or not. The accuracy of the algorithm was 95%: no false positives were found, and only four false negatives were returned (labels with a mention to purification that could not be read).

A closer review of the original label and read values found that a lack of a standardized date format incurred errors in the algorithm: different characters, such as ‘/’, ‘.’, or ‘-’ are used as separators; years are either printed with two or four digits (17 vs. 2017).

**Figure 2:**
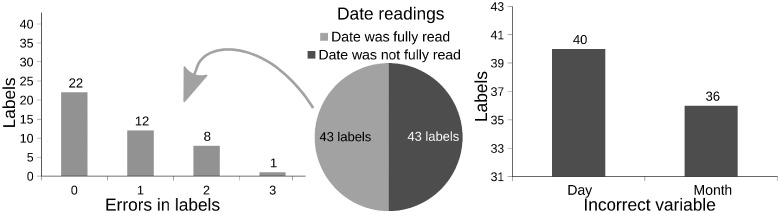
Detailed label recognition error analysis

Figure 2 presents a detailed view of the algorithm results by segregating the labels with correct and incorrect dates. The pie chart of figure 2 shows the number of labels from which the date was (in)accurately read.

The left side of figure 2 regards the labels on which the date was correctly read and shows the total number of erroneous data produced by the algorithm. More than half of those readings had no errors on the remaining variables. This graphic represents the quality of recognition of the textual data on the labels that considering the variability found on the label structure, can be considered promising.

The graph on the right presents what errors were found when reading the dates. There rate of errors in the day and month component of the date is similar, meaning that the problem with the date reading is transversal to these two components.

A comparison of the date format with the algorithm accuracy can also be done. For each type of date, the algorithm accuracy can be calculated (percentage of dates that were correctly read):

- Slash separator (dd/mm)—65% accuracy
- Hyphen Separator (dd-mm)—39% accuracy
- Dot Separator (dd.mm)—28% accuracy

## Discussion

The majority of the labels were of bivalves produced in Portugal. Of those, the large majority included extra data (not required by the food labelling law): the classified production area and whether were processed in purification facilities. This information will allow the users to better evaluate the characteristics of the production chain of the product.

Over 30% of the sampled labels come from bivalves that were produced outside of Portugal. The results show that some of these labels also include extra information. The extra information can be used by the consumer to access consumption authorization and required controls results.

The development of a mobile application to ease the access of bivalves quality controls becomes promising, because: (i) a large the number of label include the necessary information for this application to be useful (classified production area, and packaging date), (ii) information whether the product was processed in a purification unit is also present, (iii) even label of bivalves produced outside Portugal include the extra information and (iv) data from the required to controls done on the classified production areas is existent.

Nonetheless, to make it fully useful to disseminate bivalves’ quality information, a few changes should be made: (i) the food labelling laws should require the production classified area to be included in the label, (ii) authorities should make the analysis and classification areas information widely available through web services.

In parallel, with respect to bivalves produced in different countries from where they are consumed or commercialized, a global platform that would aggregate the information from all regulating entities in Europe would also be necessary. This platform could replicate the proposed for the Adriatic Sea^17^ but at a European level and make data available through web services accessible remotely. This way, any user would have access to updated information about the toxicity controls of all classified production areas Europe, either directly through the platform or through a mobile application which would be linked to the platform.

The accessibility of such mobile application could be enhanced if a label-reading algorithm (similar to the described previously) was integrated into it. The algorithm described in this study performs with an overall accuracy of 79.85%. If incorporated into a mobile application, it would ease the input of the data and speed the access to some information regarding live bivalves’ consumption safety.

Multiple factors limit the algorithm's accuracy, such as non-uniform label structure, poor quality of label printing, label degradation and poor photo illumination and focus.

The standardization of label structure would allow for a more accurate and efficient label processing, thus reducing errors. A non-variable label structure is imperative for optimal implementation and functioning of this more advanced algorithm such as those based in Machine Learning (for instance using Neural Networks) and allowing a better recovery from label damages. For example, in the sampled labels the dates written with slashes as separator were read with fewer errors.

Ideally, the food labelling laws should require labels to be printed according to a standardized structure with the clear separation and identification of all the required fields. This would, not only help automatic processing of the label, but also help the reading of that information by the consumers. Furthermore, the inclusion of additional normalized information (for instance a global identifier of the original lot and classified production areas) complemented by a proper global information system could also help solve the current traceability problem.^18^

In conclusion, this study proved that some extra information is being included in bivalves labels and that this information (classified production areas) would allow the consumers and other entities to access detailed quality control information of those products. The use of automatic label-reading algorithm integrated into a mobile application and online access to information regarding bivalve consumption safety is promising but can only be implemented with further regulation of the labels: additional required information and a more standardize label structure.
